# Enhancing Large Language Models With AI Agents for Chronic Gastritis Management: Comprehensive Comparative Study

**DOI:** 10.2196/73857

**Published:** 2025-11-13

**Authors:** Shurui Wang, Qing Ye

**Affiliations:** 1 Tongji Hospital, Tongji Medical College, Huazhong University of Science and Technology, 1095 Jiefang Ave, Wuhan, Hubei, 430000, China, +86 188-0276-3109

**Keywords:** large language models, artificial intelligence agent, AI agent, chronic gastritis, health management, retrieval-augmented generation, artificial intelligence, AI

## Abstract

**Background:**

The prevalence of chronic gastritis is high, and if not intervened in a timely manner, it may eventually lead to gastric cancer. Managing chronic gastritis essentially requires comprehensive lifestyle changes. However, the current health care environment does not support continuous follow-up by professional health care providers, making self-management a key component of postdiagnosis care. Increasingly, researchers are exploring the use of large language models (LLMs) for patient management. However, LLMs have limitations, including hallucinations, limited knowledge scope, and lack of timeliness. Artificial intelligence (AI) agents may provide a more effective solution. Nevertheless, it remains uncertain whether AI agents can effectively support postdiagnosis self-management for patients with chronic gastritis.

**Objective:**

The purpose of this study was to explore the effectiveness of AI agents in the postdiagnosis management of patients with chronic gastritis from different perspectives.

**Methods:**

In this study, we developed an agent framework for the health management of patients with chronic gastritis based on LLMs in conjunction with retrieval-augmented generation and a search engine tool. We collected real questions from patients with chronic gastritis in clinical settings and tested the framework’s performance across different difficulty levels and scenarios. We analyzed its safety and robustness and compared it with state-of-the-art models to comprehensively evaluate its effectiveness.

**Results:**

Using a dual-evaluation framework comprising automated metrics and expert manual assessments, our results demonstrated that AI agents substantially outperformed LLMs in addressing high-complexity questions (embedding average score: 82.849 for AI agents vs 77.825 for LLMs) and were particularly effective in clinical consultation tasks. Clinical evaluation of safety based on a 5-point Likert scale by physicians indicated that the safety of the agents was 4.98 (SD 0.15; 95% CI 4.96-4.99). After 30 repeated experiments, the mean absolute deviation of the AI agents in the embedding average score and BERTScore metrics were 0.0167 and 0.0387, respectively. Therefore, the safety and robustness analysis confirmed that the AI agents can produce safe, stable, and minimally variable responses. In addition, comparative results with those of advanced medical-domain LLMs (Baichuan-14B-M1 and MedGemma-27B) and general-domain LLMs (Qwen3-32B) also demonstrated that the AI agents in this study performed outstandingly in the field of chronic gastritis. Our findings underscore the superior reliability, interpretability, and practical applicability of AI agents over conventional LLMs in chronic gastritis management, offering a robust foundation for their broader adoption in health care settings.

**Conclusions:**

AI agents based on LLMs have high application value in the management of chronic gastritis. They can effectively guide patients with chronic diseases in addressing common issues, which may potentially reduce the workload of physicians and improve the quality of patient home care.

## Introduction

Chronic gastritis is one of the most common disorders in the digestive system and is also the initial phase in the progression to gastric cancer [1-3]. It is characterized by insidious onset, a protracted disease course, high prevalence, and frequent recurrence, as well as substantial health care costs, all of which significantly compromise patients’ quality of life [4]. Data show that nearly half of patients with chronic atrophic gastritis experience anxiety [5], and prolonged anxiety is also identified as a risk factor for the exacerbation of chronic gastritis. The management of chronic gastritis inherently requires comprehensive lifestyle modifications, and self-management has emerged as a critical component in chronic gastritis care. Patients can achieve not only effective symptom control but also meaningful improvement in overall health-related quality of life through systematic self-monitoring, evidence-based lifestyle adjustments, and structured psychological support, ultimately progressing toward holistic wellness.

Current constraints within the health care system render continuous postdiagnosis management by medical professionals impractical and unsustainable. The substantial heterogeneity in patient demographics further compounds clinical workload burdens. Particularly in resource-limited settings and geographically remote areas with uneven health care distribution [6,7], temporal and financial constraints make regular in-person clinical follow-ups largely unfeasible. While patient self-management presents a viable strategy to mitigate these resource limitations, the selection of appropriate self-care modalities remains paramount. Inappropriate information-seeking behaviors may lead to the acquisition of erroneous medical knowledge, potentially yielding adverse clinical outcomes. For instance, exclusive reliance on search engines for medical guidance is problematic due to the absence of professional interaction, where inaccuracies in symptom interpretation or medical misinformation may precipitate serious consequences [8]. Online consultations in the internet era provide convenience for patient inquiries. However, patients often express concerns about the protection of their personal privacy and a lack of trust in the professionalism of online services [9,10]. In addition, the uncertainty surrounding patients’ online medical needs and the availability of physicians is highly likely to intensify their hesitation and resistance toward online consultations.

Since 2018, when OpenAI introduced the first generative pretrained transformer model, GPT-1 [11], large language models (LLMs) have ushered in a golden age. In recent years, the surge in the development of LLMs has inevitably sparked transformative changes in the medical field [12]. Ayers et al [13] compared the performance of ChatGPT with that of physicians in responding to patient inquiries on social media. The results indicated that the responses generated by ChatGPT were of higher quality and received greater patient approval. In addition, LLMs are capable of maintaining continuous communication with patients around the clock, a level of availability that is nearly impossible for human responders to achieve and difficult to improve upon. Furthermore, LLMs can address sensitive questions posed by patients, which are often challenging to broach or are met with hesitation during face-to-face consultations.

However, complete reliance on LLMs also carries certain risks as the issue of hallucinations cannot be overlooked, particularly in the health care domain. As hallucinations cannot be entirely eliminated, we can strive to minimize their occurrence. Research has shown that the agent framework [14] can significantly reduce the hallucination rate [15,16]. Meanwhile, the knowledge of LLMs is derived from their pretraining data, which inherently have limitations such as restricted scope and lack of timeliness [17]. To better address the complex and dynamic management needs of patients with chronic gastritis, an agent system built upon LLMs but not confined to the existing content in the training data may offer a more effective solution. As LLMs demonstrate remarkable capabilities and attract widespread attention, an increasing number of researchers are leveraging these models to develop artificial intelligence (AI) agent systems [18-20]. Agents have been proven to possess capabilities beyond those of LLMs [21-23]. Nevertheless, whether agents can be effectively used for the management of patients with chronic gastritis remains uncertain.

Our research specifically designed a question-and-answer (Q&A) dataset for chronic gastritis and analyzed the feasibility of applying agents in the medical field. Furthermore, by categorizing health management tasks, problem complexity, and the scale of LLMs, we compared the content of responses generated by LLMs and agents. This study aimed to provide guidance for the application of AI agents in the medical field and offer practical AI tools for the postdiagnosis management of patients with chronic gastritis.

## Methods

### Study Design

The effectiveness of chronic gastritis treatment depends to some extent on the patient’s educational background, living environment, and personal habits. The actual circumstances of patients vary, leading to differences in the complexity of the questions they raise. In addition, prompts can significantly influence the performance of LLMs [24,25], and LLMs of different scales exhibit varying capabilities in processing prompts. Therefore, taking into account the characteristics of the disease, the scale of the model, and the needs of patients, we aimed to explore the following research questions:

1. In the context of chronic gastritis management, does a larger LLM lead to better response content?
2. In chronic gastritis management, with a consistent parameter size, does an agent outperform an LLM?
3. How does the response content of Q&A models change as the difficulty of the questions increases?
4. Across different health management task scenarios, does an agent consistently outperform an LLM?

**Figure 1.**
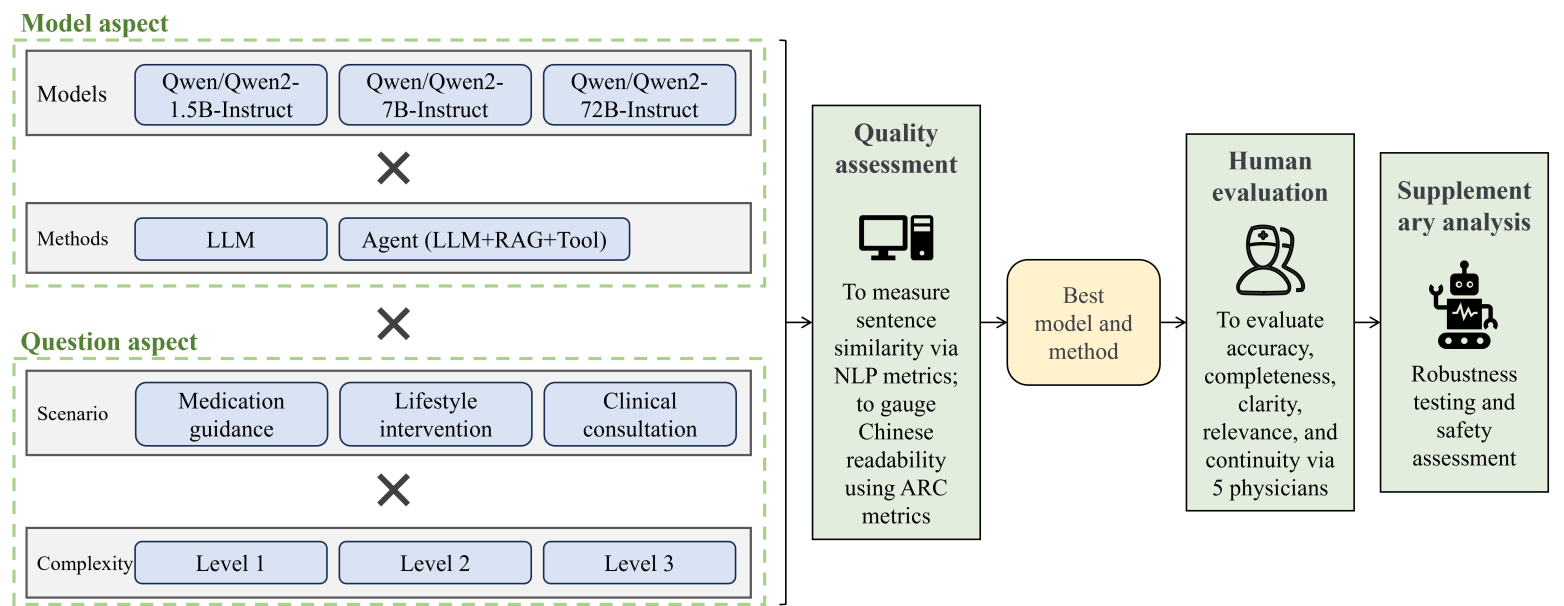
Study framework for the application of large language models (LLMs) in chronic gastritis management. ARC: Alpha Readability Chinese; NLP: natural language processing; RAG: retrieval-augmented generation.

To address the aforementioned questions, we propose the methodological framework illustrated in Figure 1. On the basis of health management task scenarios (lifestyle intervention, medication guidance, and clinical consultation) and problem complexity (level 1, level 2, and level 3; more details can be found in the Preparation of the Q&A Dataset section), questions were categorized into 9 (3 × 3) dimensions. These questions were then tested using 3 scales of LLMs and agents (using retrieval-augmented generation [RAG] and a search engine tool; more details can be found in the Use of LLMs and Agents section) on real-world problems. The responses were evaluated using multiple assessment metrics (more details can be found in the Model Evaluation section), and the performance of the models was analyzed across various aspects, including model size, methodology, task scenarios, and problem difficulty. To ensure the safety of the generated answers, we assessed all responses for safety, mitigating potential risks associated with the experimental outcomes. Finally, to enhance the robustness of the model, we conducted multiple tests on the same set of questions to confirm the stability of the results.

### Preparation of the Q&A Dataset

**Figure 2.**
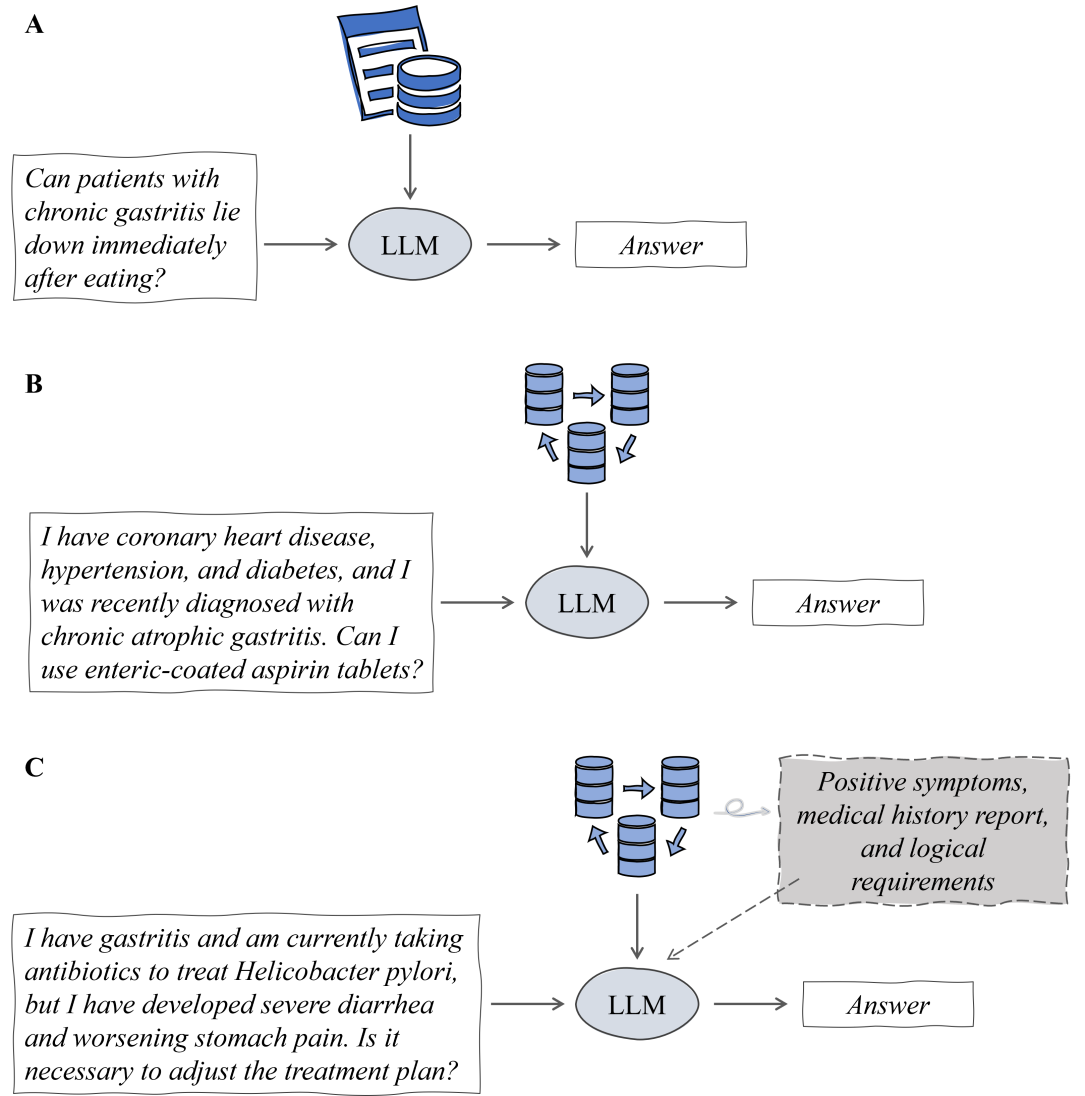
Criteria for question difficulty classification. (A) Low-difficulty questions, which can be answered based on explicit facts, primarily require the model to locate and extract relevant information. (B) Medium-difficulty questions, which require implicit facts to derive the answer, may involve information scattered across multiple segments. (C) High-difficulty questions, where no explicit answer exists in any information segment, demand complex reasoning to uncover latent information. LLM: large language model.

Q&A design was one of the most critical aspects of this study. The real-world questions selected must be representative. We classified the difficulty levels of the patient questions based on the cutting-edge methods proposed by Microsoft [26]. Microsoft’s hierarchical approach is designed to identify pathways for finding answers and is tailored for general domains, which does not fully align with the classification of problem difficulty in medical scenarios. Building on this, we collaborated with clinical experts to define 3 difficulty levels appropriate for medical scenarios: low-difficulty questions (level 1; based on explicit facts), medium-difficulty questions (level 2; based on implicit facts), and high-difficulty questions (level 3; requiring reasonable inference). Figure 2 illustrates the rules for categorizing question difficulty.

### Use of LLMs and Agents

As the models in the Qwen series have been proven to have excellent application performance [27,28], we selected the “instruct” models from the Qwen2 series with model sizes of 1.5B, 7B, and 72B as the foundational models (Qwen-1.5B, Qwen-7B, and Qwen-72B models) [29]. In 2020, Lewis et al [30] formally proposed and named the RAG framework, marking the birth of a new paradigm. Research has shown that, with the support of external knowledge, “instruct” models can generate more accurate and higher-quality responses [30,31]. Using domain-specific knowledge and external tools, we applied instruction prompting to these models to improve their specialization in the management of chronic gastritis. In addition, an agent was constructed based on the LLM framework, enabling the models to provide more professional responses to questions from patients with chronic gastritis. To enhance the capabilities of the agent, we used RAG and integrated search engine tools to prepare contextual information. The detailed process of RAG and the visualized integration of its components, as well as the prompt templates, are shown in Multimedia Appendix 1. Our preparatory work can be divided into the following components:

- Knowledge base: in light of the updates in clinical techniques, we conducted a search in the *National Medical Journal of China* for Chinese expert consensuses and clinical guidelines published from 2020 to 2024 using “gastritis” as the keyword. Subsequently, we invited experts to manually screen and select 18 articles related to chronic gastritis. Using the zh_core_web_sm model, we segmented the long texts into sentences and further divided these sentences into chunks using a sliding window approach. Each chunk contained 20 sentences, with an overlap of 5 sentences between adjacent chunks to maintain contextual coherence. Subsequently, the all-MiniLM-L6-v2 model was used to map these chunks into a vector space, converting them into embedding vectors. Finally, using the Euclidean distance as the similarity metric, we created a Faiss index and added the embedding vectors to this index to enable rapid retrieval of the most similar vectors for a given query text.
- Agent tools: a defining feature of an agent, as opposed to a stand-alone LLM, is its ability to flexibly use external tools. In this process, we invoked the Google Search application programming interface to retrieve URLs and content summaries related to the input query. The summary texts were then ranked based on relevance to produce the final search results.
- Agent (with RAG and search tools): on the basis of the Faiss index and L2 distance, we retrieved the 5 most relevant text fragments from the knowledge base. The Google Search application programming interface sorted the organic results according to its internal ranking algorithm, and the 5 most relevant web page snippets were directly output. These 10 text fragments were then passed to the model as reference materials, whereas the decision of whether to use this reference content was autonomously made by the agents. Three agents were constructed based on the Qwen-1.5B, Qwen-7B, and Qwen-72B models. These agents shared identical network parameters, which were configured as follows: the maximum number of tokens was set to 800, the temperature was set to 0.4, the nucleus sampling parameter was set to 0.8, the truncation parameter was set to 5, and the frequency penalty was set to 0.5.

### Model Evaluation

Relying on a single perspective for evaluation metrics may lead to biased results. To address this, we used 3 types of evaluation methods: natural language processing–based automatic evaluation metrics, manual scoring, and Chinese lexical evaluation metrics. The automatic evaluation metrics compared the system-generated answers with the standard answers (more details are provided in the Preparation of the Q&A Dataset section) to derive evaluation results. Among these, the embedding average score and BERTScore use cosine similarity between vectors to calculate the relevance to the standard answers, which has proven to be an effective approach [32-34]. In light of the unique nature of medical scenarios, we also designed a Likert scale and organized a panel comprising 5 clinical experts in relevant fields (including 1 certified clinical nutritionist), all of whom have over 10 years of work experience, to manually score the model-generated answers based on the following criteria [35]:

- Accuracy: the response contained specific and precise information rather than general or generic information.
- Completeness: the response covered all relevant medical information and details, with no omission of critical content.
- Clarity: the response was clearly expressed, easy to understand, readable, and free of ambiguity.
- Relevance: the response was closely related to the question, providing useful information and recommendations that aligned closely with the patient’s needs.
- Continuity: the response maintained coherent and consistent phrasing throughout, with no logical jumps or inconsistencies.

The experts scored the responses generated by each model using each method (3 × 2 × 63) on a scale ranging from 1 to 5, where a higher score indicated better performance. In addition, we used a specialized evaluation framework designed for Chinese text to assess the richness and clarity of syntax and vocabulary [36]. This framework was originally developed for general Chinese contexts, but its evaluation metrics can be adapted and interpreted more granularly for medical scenarios:

- Lexical richness: the entropy values of all the words were calculated. The higher the entropy value, the greater the uncertainty associated with the words used in the text. This indicates a more diverse vocabulary, which in turn increases the reading difficulty level of the text.
- Syntactic richness: the entropy values of all the dependencies in the text were calculated. The higher the entropy value, the greater the uncertainty in the text’s dependency relationships or syntactic structure. This suggests a more complex and varied syntax, which in turn makes the text more difficult to read.
- Semantic clarity: the semantic clarity value was calculated based on the skewness of the topic distribution probability extracted through latent Dirichlet allocation topic modeling [37]. The higher this value, the more concentrated the text’s topics represented by nouns, resulting in clearer semantics.
- Semantic noise: the semantic noise value was calculated through the kurtosis of the topic distribution probability extracted through latent Dirichlet allocation topic modeling [37]. The higher this value, the more the text’s topics represented by nouns are skewed toward unimportant topics, thereby increasing the semantic noise.
- Semantic richness: on the basis of the research by Lee et al [37], the semantic richness of the text was calculated by summing the probabilities of the occurrence of nouns in the text. The higher the value, the richer the topics of the text, and the lower its readability.

### Ethical Considerations

This study aimed solely to evaluate the quality of responses generated by an AI agent for chronic gastritis queries and was therefore classified as nonhuman-participant research; institutional review board approval and full ethics review were waived. All patient questions were gathered during routine clinical operations, and no identifiable personal information was collected. Consequently, informed consent was not required and no compensation was provided. All analyses were performed on a secure server to safeguard the privacy and confidentiality of the questions submitted by patients.

## Results

### Influence of Model Scales and Methods on Response Content

#### Model Scale Comparison

**Figure 3.**
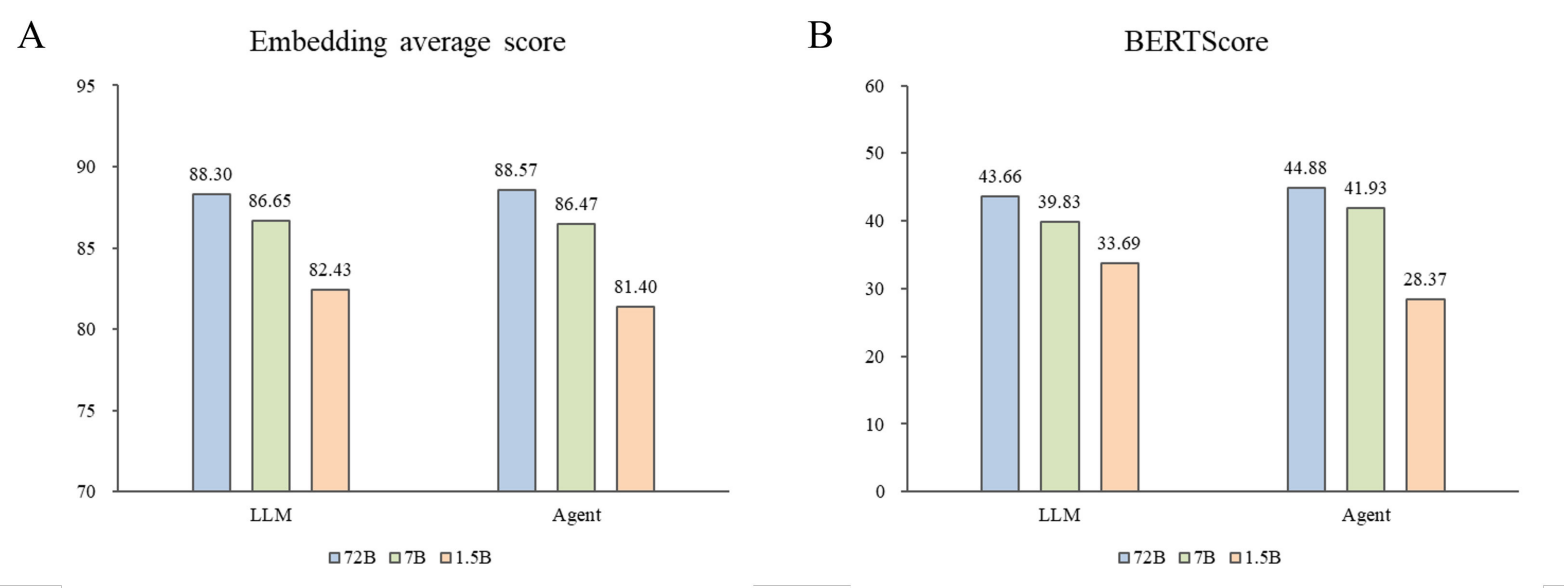
Statistical differences in evaluation indicators under different model parameters. Median differences were compared using bar charts. LLM: large language model.

The number of parameters is one of the primary factors influencing the performance of LLMs [38]. The results shown in Figure 3, Table 1, and Figure S3 in Multimedia Appendix 1 demonstrate that changes in model scales have a positive impact on the response content of LLMs. However, as the number of parameters increased, the improvement effect gradually diminished. Specifically, there was no statistically significant difference in response content between base models with 7B and 72B parameters. This phenomenon may be attributed to 2 reasons. First, general-purpose LLMs are suitable for answering questions in broad domains, but their actual performance in specialized fields such as medicine remains uncertain [39,40]. Second, the 7B model has already reached a bottleneck in understanding medical questions, and as our questions were all within the specialized medical domain, merely increasing the number of parameters did not significantly enhance the quality of the response content. Therefore, we further tested the relationship between model scale and response content using an agent. The results still indicated that the Qwen-72B model was the optimal choice for a health management Q&A model focused on chronic gastritis.

**Table 1.** Mann-Whitney *U* test results for evaluation indicators across different model parameters.

Indicator	*U* value	*Z* value	*P* value
Embedding average score			
LLM^a^ 1.5B vs 7B	1376	−2.969	.003
LLM 1.5B vs 72B	1336	−3.164	.002
LLM 7B vs 72B	1840	−0.705	.48
Agent 1.5B vs 7B	1165	−3.999	<.001
Agent 1.5B vs 72B	873	−5.423	<.001
Agent 7B vs 72B	1534	−2.198	.03
BERTScore			
LLM 1.5B vs 7B	1203	−3.813	<.001
LLM 1.5B vs 72B	1152	−4.062	<.001
LLM 7B vs 72B	1781	−0.993	.32
Agent 1.5B vs 7B	730	−6.121	<.001
Agent 1.5B vs 72B	524	−7.126	<.001
Agent 7B vs 72B	1484	−2.442	.01

a LLM: large language model.

#### Comparison of LLMs and Agents

**Figure 4.**
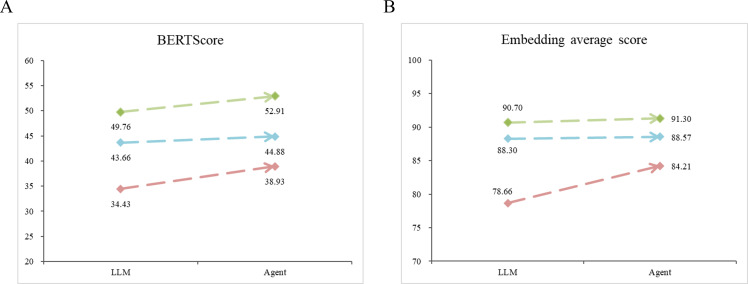
Method comparison: large language model (LLM) and agent. The blue line represents the median trend line, whereas the green and red lines represent the quartile trend lines.

Using the Qwen-72B model as the base model, we compared the response content of the LLM and the agent. The trend chart (Figure 4) shows that the response quality of the agent had an upward trend compared with that of the LLM. The linguistic evaluation results (Table 2) show that the responses generated by the agent had higher values in lexical richness, syntactic richness, semantic noise, and semantic richness and a lower value in semantic clarity. These Chinese evaluation indicators are comparative metrics that do not focus on individual results and do not have a defined range of values. This indicates that the responses generated by the agent were more complex in terms of vocabulary and syntax, had a higher reading difficulty level, and covered a broader range of topics compared to the responses generated by the LLM. This demonstrates that the agent not only outperformed the LLM in terms of alignment with the standard answers but also exceeded the LLM in terms of breadth of thinking and domain-specific expertise. This is one concrete example demonstrating the effectiveness of using the tool (Google search engine).

**Table 2.** Comparison of a large language model (LLM) and agent in Chinese-language dimensions.

	LLM 72B	Agent 72B
Lexical richness	4.0707	4.2950
Syntactic richness	2.1529	2.1911
Semantic clarity	0.0707	0.0643
Semantic noise	5.8923	6.3081
Semantic richness	0.2411	0.2520

### Influence of Question Difficulty and Medical Contexts on Answer Quality

#### Comparison of Response Content Across Different Difficulty Levels

**Figure 5.**
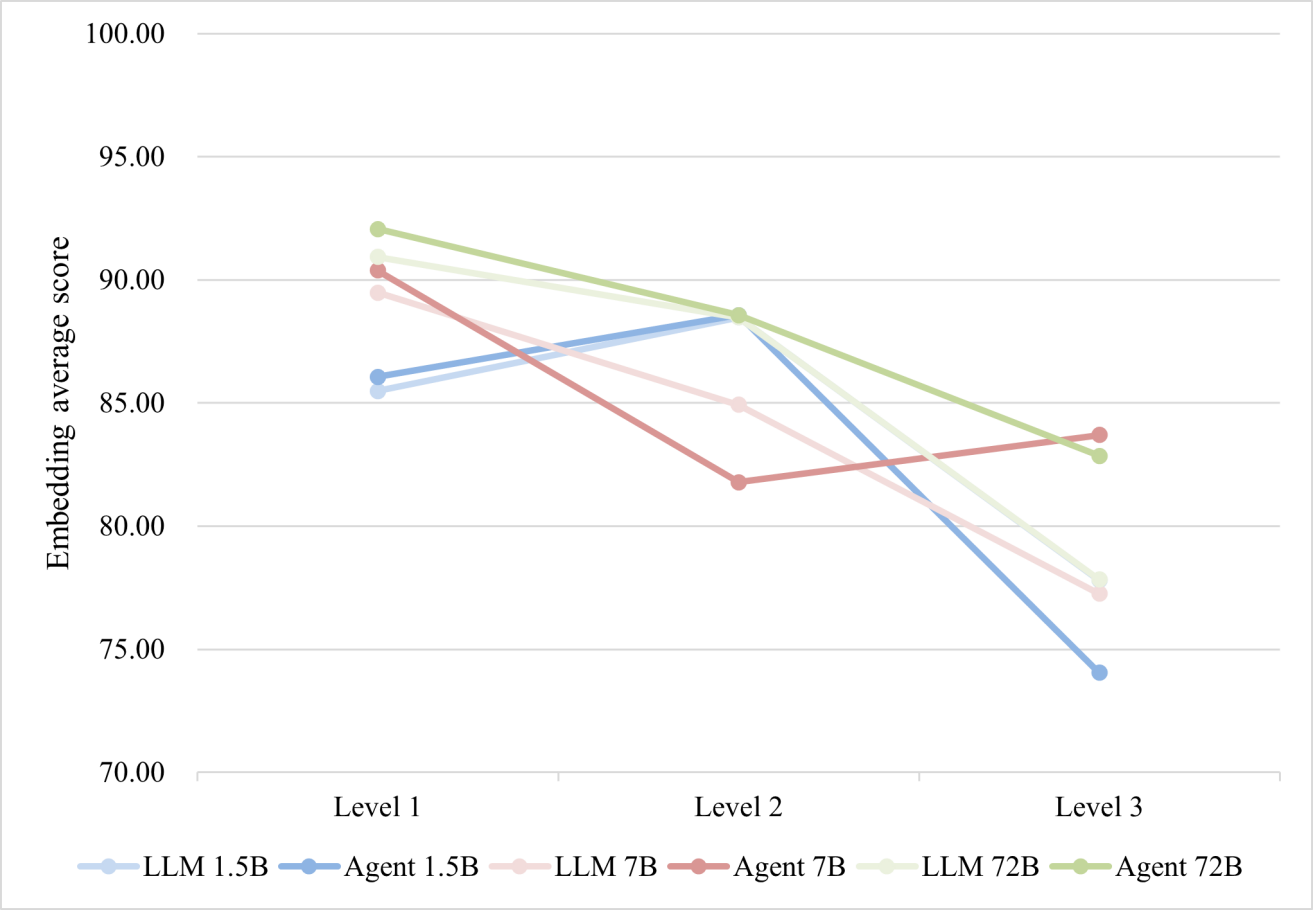
The trend in the impact of question complexity on answer quality. The points on the line represent the median of the embedding average score for all questions under that category. LLM: large language model.

Figure 5 shows the results stratified by difficulty level, which align with the previous conclusions: larger models outperformed smaller models, and the agents surpassed the LLMs. As question difficulty increased, the similarity between response content and reference answers showed a declining trend. Mann-Whitney *U* tests on the Embedding Average Scores of the 72B-Agent across difficulty levels revealed that the observed decreases from level 1 to level 2 and from level 2 to level 3 were statistically significant (*U* value=141.000, *Z* value=–2.000, *P*=.046; *U* value=121.000, *Z* value=–2.503, *P*=.01). Smaller models exhibited instability, which may be attributed to the limitations of LLMs in specialized domains, insufficient contextual comprehension by smaller models, and the negative impact of irrelevant information [41]. When domain-specific knowledge obtained through RAG and search engine tools was input into smaller agents, these models struggled to accurately understand the relevant information, resulting in noisier and less reliable outputs.

#### Comparison of Response Content From the Qwen-72B Model Across Different Health Management Tasks

**Figure 6.**
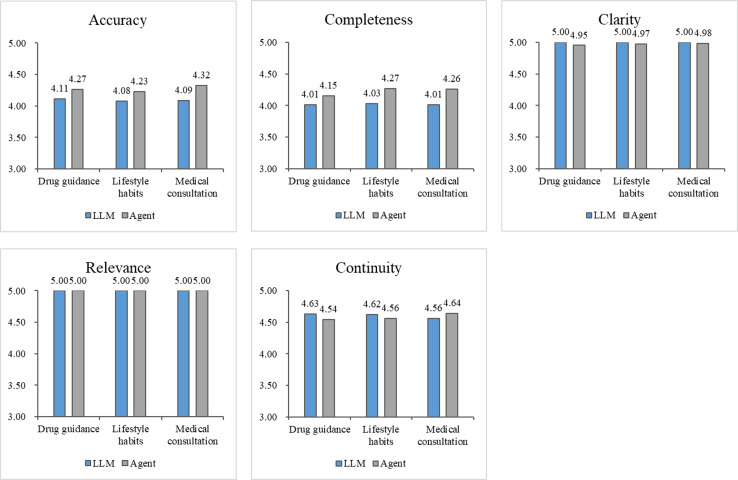
Comparison of different health management tasks. On the basis of the statistical results of human evaluation, the mean score assigned by all experts to all questions under a specific category of health management tasks was calculated. LLM: large language model.

The manually assigned scores for the Qwen-72B model were aggregated and analyzed from the perspective of health management tasks (Figure 6). On the basis of the evaluation results of multiple experts, the clarity and coherence of the agent’s responses were slightly inferior to those of the LLM, which was consistent with the previous findings. This is attributed to the agent’s replies being more specialized, covering a broader range of topics, and having a higher reading difficulty level. Overall, across the 3 health management task scenarios, the agent demonstrated superior application performance compared to the LLM, with higher accuracy and completeness in its responses. Correspondingly, the increased reading difficulty level placed greater demands on patients’ comprehension abilities. In medication guidance and clinical consultation tasks, the aggregate accuracy score of the agent exceeded that for lifestyle intervention tasks, indicating that the agent could more precisely understand questions with relatively fixed answers and generate targeted responses. However, the completeness of the agent’s responses for medication guidance tasks was slightly inferior to that for lifestyle intervention and clinical consultation tasks. This suggests that the knowledge base and search engine tools provided to the agent need to be further enhanced in terms of drug-related knowledge to improve its capability in medication guidance.

### Comprehensive Evaluation of Q&A Models

The use of AI tools in medical scenarios carries unique considerations as medical assistance tools must meet stringent quality assurance standards. Figure 6 and Multimedia Appendix 1 show the expert ratings for the dimensions of health management and model characteristics, respectively. As the parameter size of the foundational model increased, expert ratings also gradually increased. However, agents based on smaller models, due to their limited ability to comprehend complex contexts, exhibited poorer performance compared to LLMs of equivalent size. When the size of the foundational model increased, the superiority of agents over LLMs became significantly more pronounced, whereas the increased reading difficulty level resulting from the enhanced professionalism of the responses also became evident. Multimedia Appendix 1 shows the results of the ablation experiment, indicating that the improvements in the agents stemmed from the synergistic effects of RAG and search tools.

A comparative analysis was conducted between the model with the strongest comprehensive capabilities (agent 72B) and 3 newly released LLMs in 2025: 2 medical LLMs, Baichuan-14B-M1 [42] and MedGemma-27B [43], and the 32B model from the Qwen3 series [44]. This study revealed that Baichuan-14B-M1 outperformed larger-scale models such as Qwen2.5-72B and Llama 3-70B in the medical domain [42]. Meanwhile, MedGemma-27B surpassed models such as BioMistral-7B-DARE and OpenBioLLM-70B [43]. As shown in Table 3, the BERTScore and embedding IQRs computed over 63 questions indicate that the agent 72B achieved the highest performance, surpassing both the latest general-domain LLMs and medical-domain LLMs on chronic gastritis health management tasks.

**Table 3.** Comparison of state-of-the-art models.

Model	BERTScore, median (IQR)	Embedding average score, median (IQR)
Baichuan-14B-M1	43.142 (37.151-48.233)	86.024 (80.914-89.266)
MedGemma-27B	40.448 (35.130-47.228)	86.887 (82.274-89.869)
Qwen3-32B	40.650 (33.372-47.909)	85.626 (81.589-89.156)
Agent 72B	44.884 (38.934-52.909)	88.574 (84.211-91.298)

### Safety Analysis

In a survey of 11,004 adults, 6602 (60.0%) reported feeling uneasy about health care professionals using AI-assisted tools, underscoring the critical need to establish patient trust [45]. Ensuring safety is key to achieving this goal. Using the Likert scale mentioned previously, experts evaluated the safety (harmful outputs such as the fabrication of false information, dissemination of erroneous data, presence of biases, associated risks, alterations, and plagiarism) of the models’ outputs [46-48]. As shown in Table 4, base models with a size larger than 7B demonstrated sufficient capability to generate relatively safe responses for patients. Multimedia Appendix 1 presents a representative error case generated by agent 7B accompanied by expert commentary.

**Table 4.** Safety assessment results.

Model	Safety score (1-5; SD; 95% CI)
LLM^a^ 1.5B	4.85 (0.62; 4.79-4.92)
Agent 1.5B	4.42 (1.06; 4.31-4.54)
LLM 7B	4.98 (0.13; 4.97-5.00)
Agent 7B	4.95 (0.27; 4.92-4.98)
LLM 72B	4.95 (0.21; 4.93-4.98)
Agent 72B	4.98 (0.15; 4.96-4.99)

a LLM: large language model.

### Robustness Analysis

The stability of the responses generated by the Q&A model was also a critical factor in determining its suitability for clinical applications. To further analyze the robustness of the agent, we preserved the original parameters and conducted supplementary evaluations based on the preconfigured agent. We randomly selected 1 patient question from each difficulty level and each type of health management task, resulting in a total of 9 questions. Each of these 9 questions was input into the agent 30 times, yielding 270 (9 × 30) outputs. For each output, we calculated the embedding average score, BERTScore, and the corresponding mean absolute deviation (MAD). The results showed that the MAD for the embedding average score was 0.0167 and the MAD for the BERTScore was 0.0387. These findings indicate that the agent based on the Qwen-72B model performed with high stability, exhibiting minimal random fluctuations and demonstrating strong robustness.

## Discussion

### Principal Findings

The evidence from this study demonstrates that LLM-based agents possess considerable potential in the management of chronic gastritis. The responses generated by agents were generally superior to those produced by LLMs, effectively addressing the limitations of LLMs in handling high-complexity questions. Moreover, agents exhibited higher safety and stability and were capable of outperforming LLMs in the cutting-edge medical domain.

This study presents a comprehensive evaluation of LLM-based agents’ effectiveness in chronic gastritis management across heterogeneous clinical scenarios and different scales. Our multimetric analysis revealed 4 critical findings that advance the understanding of AI-driven medical decision support systems. The first finding relates to model scaling effects. The quality of responses for chronic gastritis exhibited progressive enhancement with increasing base model sizes. Larger architectures (eg, Qwen-72B) demonstrated superior medical information processing capabilities through improved semantic comprehension and clinical reasoning. Conversely, smaller models showed inherent limitations in effectively integrating medical knowledge derived from RAG and search tools, resulting in suboptimal domain-specific performance. This parameter-performance correlation aligns with neural scaling laws while highlighting critical capacity thresholds for medical AI applications. The second finding relates to agent versus base LLM performance. When using base models with larger parameters (eg, Qwen-72B), the agents demonstrated better performance compared to the LLMs, particularly in terms of answer accuracy and completeness. However, due to the agents’ stronger medical expertise in generating responses, as well as their higher lexical and syntactic complexity, the readability of their answers tended to be lower than that of the LLMs’ answers. The third finding relates to the impact of problem complexity. As the difficulty of the questions increased, the response quality of both Q&A models showed a declining trend. However, overall, the agents outperformed the LLMs across all 3 difficulty levels, particularly in handling high-difficulty questions, where the agents significantly compensated for the LLMs’ shortcomings in the medical domain. The fourth finding relates to performance on different tasks. When addressing different health management task scenarios, the agents demonstrated statistically significant superiority over the LLMs in both accuracy and completeness of responses, with the most notable improvement observed in clinical consultation scenarios. Lifestyle intervention questions were subjective and did not have a single standard answer, resulting in limited improvement from RAG and search tools. Drug information is complex and varied, as medications produced by different manufacturers may have differences in use details, and the current information is insufficient to fully cover all potential patient inquiries. Despite these challenges, the agents still exhibited higher practical value in chronic gastritis management compared to the LLMs.

This study has both theoretical and practical contributions. On the theoretical side, first, this study demonstrated that LLM-based agents outperform generalized LLMs in multiple scenarios of chronic gastritis management. Second, this study demonstrated that the larger the parameters of either the base model or the LLM-based agents, the better the performance of the model, even though the quality of the answers tended to decrease as the difficulty of the questions increased. Finally, this study demonstrated that the LLM-based agents had better performance in multiple scenarios of chronic gastritis management. On the practical side, this study identified the value of LLM-based agents in chronic disease management. By using real-world problems and multiscenario chronic disease management tasks, we validated the capabilities of these agents. This provides a reliable LLM-driven approach to the management of chronic gastritis, paves the way for future LLM-based chronic disease management, and provides a more flexible form of counseling for patients.

There are some limitations to our study. First, we only used textual data, whereas data in the clinical setting also include modalities such as temporal, image, and video data. Future research could use multimodal data to evaluate the performance of LLMs in chronic disease management. Second, due to the privacy management requirements of health care data, we only used privately deployed models, which generally have a small number of participants. Future research can further explore models with a larger number of parameters, such as the GPT-4 family of models. Third, we did not use medical bigram models such as HuatuoGPT [49]. This is because these models have been fine-tuned using the medical corpus, and the unknown corpus may affect our evaluation and cause cognitive bias in specific scenarios. Fourth, the hallucination issue in LLMs cannot be overlooked. While agents can reduce the probability of hallucinations, to prevent potential adverse impacts on health care applications, we recommend incorporating a hallucination threshold control mechanism in future studies. This system would automatically suspend operations and initiate retraining when the false positive rate exceeds predefined safety thresholds. It should be noted that these findings are based solely on Chinese-language data. Future research could validate these results across different linguistic and cultural contexts. In addition, patient surveys and randomized controlled trials could be conducted to investigate factors influencing patients’ use of AI tools in real clinical settings. Such studies would provide further guidance for enhancing the effectiveness of chronic gastritis self-management tools.

### Conclusions

In this study, we compared the effectiveness of agents and LLMs on a chronic disease Q&A dataset across different levels of difficulty and various scenarios. Our multiperspective evaluation results show that the responses generated by the agents were often preferred over those of the LLMs due to their higher embedding average score, BERTScore, accuracy, and completeness values, as well as their higher values in other metrics. The LLM-based agents demonstrated advantages across different difficulty levels, particularly addressing the shortcomings of LLMs in handling high-difficulty questions. Furthermore, the LLM-based agents exhibited varying application effectiveness in different health management task scenarios, proving more suitable for questions with relatively fixed answers. Compared with state-of-the-art general-purpose models and medical-domain large models, the 72B agent further demonstrates its professional competence in the health management of chronic gastritis. The robustness and safety analyses we conducted explored the stability of the agents’ responses and their safety for clinical application. The results of this study suggest that LLM-based agents have high value for application in the management of chronic gastritis and that they are effective in guiding patients with chronic diseases in solving common problems, thereby potentially reducing clinicians’ workload and improving the quality of patients’ home care.

## Supplementary material

10.2196/73857 Multimedia Appendix 1 Supplementary tables and figures.

## Abbreviations

AI: artificial intelligence
LLM: large language model
MAD: mean absolute deviation
Q&A: question and answer
RAG: retrieval-augmented generation
