# Supplementary material for: Enhancing Large Language Models With AI Agents for Chronic Gastritis Management: Comprehensive Comparative Study
Source: JMIR Med Inform. 2025 Nov 13;13:e73857. doi: 10.2196/73857 (PMC12617012; doi:10.2196/73857)
Supplement: Multimedia Appendix 1 [file medinform-v13-e73857-s001.docx]

# **Appendix A**


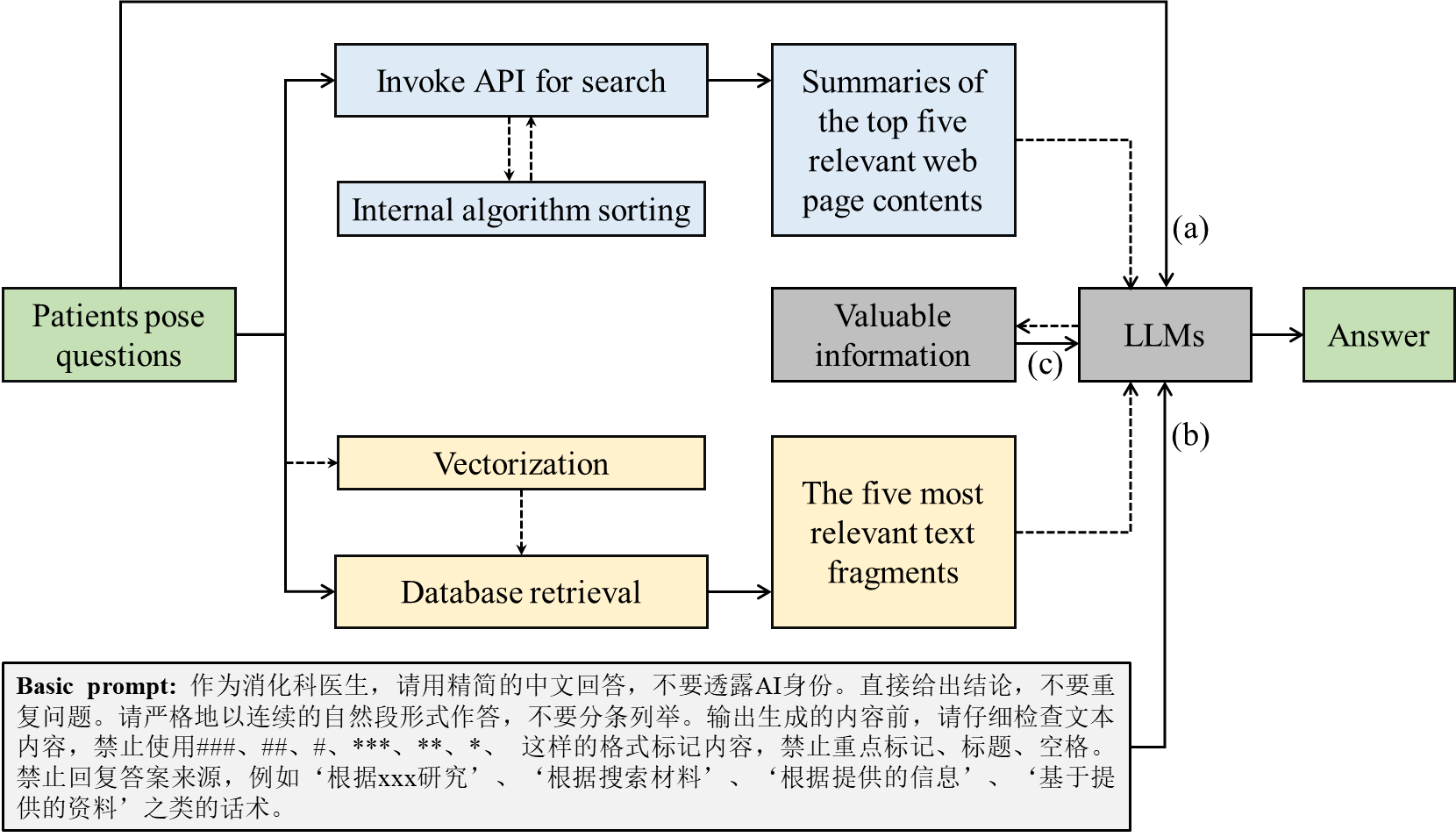


**Figure A.7. System Architecture Diagram.** It encompasses the entire process of querying, retrieving, integrating, constructing prompts, and generating responses.


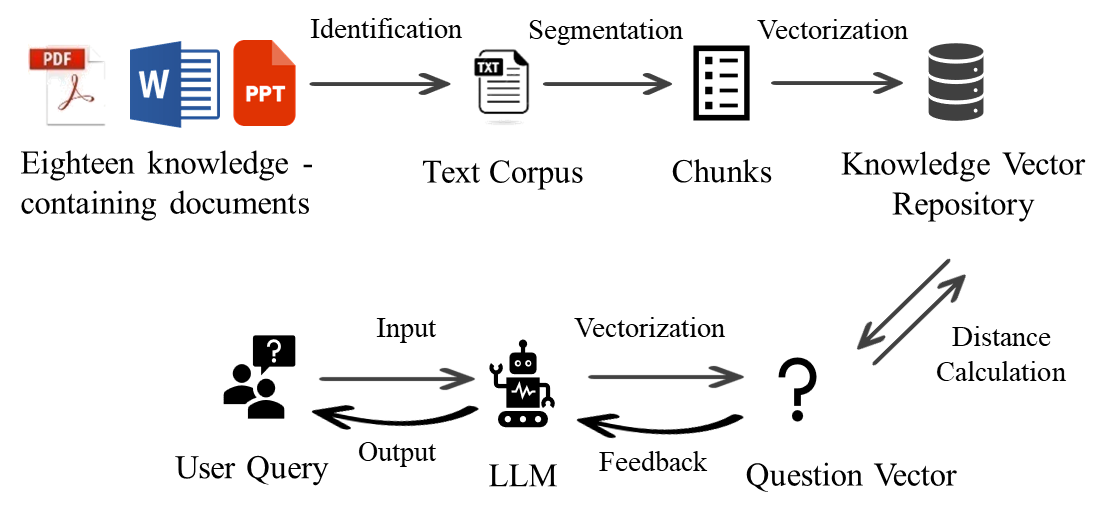


**Figure A.8. Retrieval-Augmented Generation (RAG) Flowchart.**

# **Appendix B**

**Prompt Template for LLMs:**

作为消化科医生，请用精简的中文回答，不要透露AI身份。直接给出结论，不要重复问题。请严格地以连续的自然段形式作答，不要分条列举。输出生成的内容前，请仔细检查文本内容，禁止使用###、##、#、***、**、*、 这样的格式标记内容，禁止重点标记、标题、空格。禁止回复答案来源，例如‘根据xxx研究’、‘根据搜索材料’、‘根据提供的信息’、‘基于提供的资料’之类的话术。患者问题：[Query]

**Prompt Template for Agents:**

作为消化科医生，请用精简的中文回答，不要透露AI身份。直接给出结论，不要重复问题。请严格地以连续的自然段形式作答，不要分条列举。输出生成的内容前，请仔细检查文本内容，禁止使用###、##、#、***、**、*、 这样的格式标记内容，禁止重点标记、标题、空格。禁止回复答案来源，例如‘根据xxx研究’、‘根据搜索材料’、‘根据提供的信息’、‘基于提供的资料’之类的话术。患者问题：[Query]

参考内容不一定与问题完全相关，若参考内容对问题没有贡献，可以考虑无视。如果参考部分内容，请流畅地把相关内容加入到你的回答中，参考信息如下：[Knowledge]

# **Appendix C**


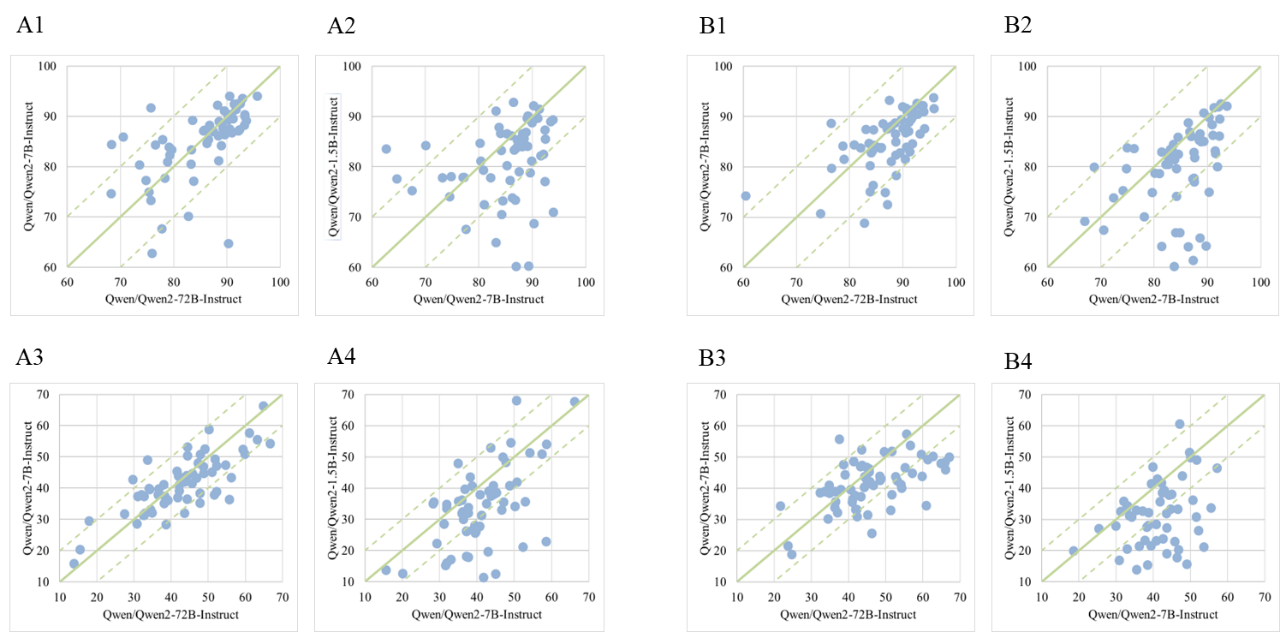


**Figure C.9. Pairwise comparisons of answer quality under model parameters.** A1-A2 represent the performance of LLMs evaluated using the Embedding Average Score, while A3-A4 represent the performance of LLMs evaluated using BERTScore. Similarly, B1-B2 represent the performance of Agents evaluated using the Embedding Average Score, and B3-B4 represent the performance of Agents evaluated using BERTScore. The points in the graph indicate the computed evaluation metric values for the same question across different model parameters. The solid green line represents the equivalence boundary, while the dashed lines indicate boundaries where the metric values differ by ±10.

# **Appendix D**


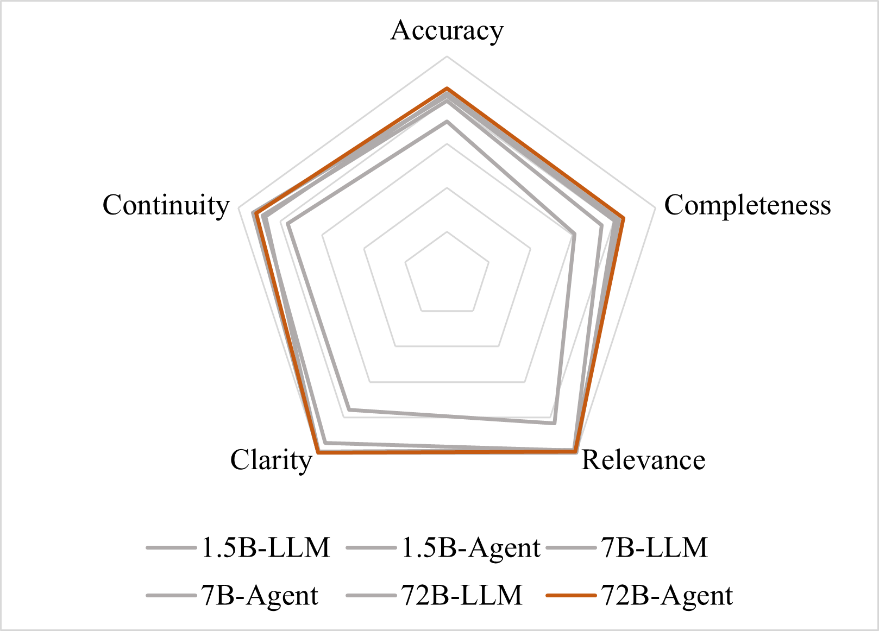


**Figure D.10. Radar chart of human evaluation results.**

**Table D.5. Result of human evaluation.**

| Evaluator | | Accuracy | Completeness | Relevance | Clarity | Continuity |
| --- | --- | --- | --- | --- | --- | --- |
| **1.5B LLM** | | | | | | |
|  | 1 | 3.98(3.88-4.09) | 3.71(3.55-5.00) | 4.73(4.56-4.90) | 4.40(4.27-4.53) | 4.40(4.27-4.53) |
|  | 2 | 4.00(3.89-4.11) | 3.71(3.55-3.88) | 4.94(4.88-5.00) | 4.73(5.54-4.92) | 4.43(4.30-4.56) |
|  | 3 | 4.00(3.89-4.11) | 3.73(3.57-3.89) | 4.94(4.88-5.00) | 4.73(4.56-4.90) | 4.43(4.30-4.56) |
|  | 4 | 3.98(3.85-4.11) | 3.68(3.50-3.87) | 4.94(4.88-5.00) | 4.68(4.49-4.87) | 4.44(4.31-4.58) |
|  | 5 | 3.97(3.84-4.09) | 3.70(3.52-3.88) | 4.90(4.83-4.98) | 4.70(4.51-4.89) | 4.38(4.25-4.51) |
|  | Pooled | 3.99(3.94-4.04) | 3.71(3.63-3.79) | 4.71(4.63-4.80) | 4.93(4.90-4.96) | 4.42(4.36-4.47) |
| **1.5B Agent** | | | | | | |
|  | 1 | 3.52(3.28-3.77) | 3.03(2.68-3.38) | 3.81(3.42-4.20) | 4.14(3.83-4.45) | 3.81(3.53-4.09) |
|  | 2 | 3.51(3.27-3.75) | 3.05(2.71-3.39) | 4.17(3.86-4.49) | 3.79(3.40-4.19) | 3.79(3.52-4.07) |
|  | 3 | 3.51(3.27-3.75) | 3.06(2.72-3.40) | 4.19(3.89-4.49) | 3.79(3.41-4.18) | 3.81(3.56-4.06) |
|  | 4 | 3.49(3.25-3.73) | 3.05(2.71-3.38) | 4.17(3.87-4.48) | 3.79(3.41-4.18) | 3.83(3.58-4.07) |
|  | 5 | 3.51(3.27-3.75) | 3.10(2.77-3.42) | 4.19(3.90-4.48) | 3.76(3.38-4.14) | 3.86(3.61-4.10) |
|  | Pooled | 3.51(3.40-3.61) | 3.06(2.91-3.21) | 3.79(3.62-3.96) | 4.17(4.04-4.31) | 3.82(3.70-3.93) |
| **7B LLM** | | | | | | |
|  | 1 | 4.08(4.01-4.15) | 4.08(4.01-4.15) | 5.00(5.00-5.00) | 5.00(5.00-5.00) | 4.67(4.55-4.78) |
|  | 2 | 4.11(4.03-4.19) | 4.11(4.03-4.19) | 5.00(5.00-5.00) | 4.98(4.95-5.02) | 4.62(4.50-4.74) |
|  | 3 | 4.11(4.03-4.19) | 4.10(4.02-4.17) | 5.00(5.00-5.00) | 5.00(5.00-5.00) | 4.65(4.53-4.77) |
|  | 4 | 4.10(4.02-4.17) | 4.11(4.03-4.19) | 5.00(5.00-5.00) | 5.00(5.00-5.00) | 4.67(4.55-4.78) |
|  | 5 | 4.06(4.00-4.12) | 4.10(4.02-4.17) | 5.00(5.00-5.00) | 4.98(4.95-5.00) | 4.63(4.52-4.75) |
|  | Pooled | 4.09(4.06-4.12) | 4.10(4.07-4.13) | 4.99(4.98-5.00) | 5.00(5.00-5.00) | 4.65(4.59-4.70) |
| **7B Agent** | | | | | | |
|  | 1 | 4.16(4.07-4.25) | 4.08(4.00-4.16) | 4.97(4.92-5.00) | 4.94(4.88-5.00) | 4.38(4.26-4.50) |
|  | 2 | 4.16(4.07-4.25) | 4.14(4.05-4.24) | 4.92(4.85-4.99) | 4.97(4.92-5.00) | 4.32(4.20-4.43) |
|  | 3 | 4.16(4.07-4.25) | 4.14(4.06-4.23) | 4.94(4.88-5.00) | 4.97(4.92-5.00) | 4.38(4.26-4.50) |
|  | 4 | 4.17(4.08-4.27) | 4.13(4.03-4.22) | 4.90(4.83-4.98) | 4.97(4.92-5.00) | 4.35(4.23-4.47) |
|  | 5 | 4.14(4.06-4.23) | 4.13(4.03-4.22) | 4.92(4.85-4.99) | 4.97(4.92-5.00) | 4.32(4.20-4.43) |
|  | Pooled | 4.16(4.12-4.20) | 4.12(4.08-4.16) | 4.97(4.95-4.99) | 4.92(4.89-4.95) | 4.35(4.30-4.40) |
| **72B LLM** | | | | | | |
|  | 1 | 4.10(4.02-4.17) | 4.03(3.99-4.08) | 5.00(5.00-5.00) | 5.00(5.00-5.00) | 4.59(4.46-4.71) |
|  | 2 | 4.11(4.03-4.19) | 4.00(4.00-4.00) | 5.00(5.00-5.00) | 5.00(5.00-5.00) | 4.62(4.50-4.74) |
|  | 3 | 4.08(4.01-4.15) | 4.02(3.98-4.05) | 5.00(5.00-5.00) | 5.00(5.00-5.00) | 4.59(4.46-4.71) |
|  | 4 | 4.08(4.01-4.15) | 4.00(4.00-4.00) | 5.00(5.00-5.00) | 5.00(5.00-5.00) | 4.62(4.50-4.74) |
|  | 5 | 4.10(4.02-4.17) | 4.03(3.99-4.08) | 5.00(5.00-5.00) | 5.00(5.00-5.00) | 4.60(4.48-4.72) |
|  | Pooled | 4.09(4.06-4.12) | 4.02(4.00-4.03) | 5.00(5.00-5.00) | 5.00(5.00-5.00) | 4.60(4.55-4.66) |
| **72B Agent** | | | | | | |
|  | 1 | 4.32(4.20-4.43) | 4.25(4.15-4.36) | 5.00(5.00-5.00) | 4.95(4.90-5.00) | 4.63(4.52-4.75) |
|  | 2 | 4.25(4.15-4.36) | 4.21(4.11-4.31) | 4.97(4.92-5.00) | 5.00(5.00-5.00) | 4.59(4.46-4.71) |
|  | 3 | 4.24(4.13-4.34) | 4.22(4.12-4.33) | 4.97(4.92-5.00) | 5.00(5.00-5.00) | 4.56(4.43-4.68) |
|  | 4 | 4.27(4.16-4.38) | 4.19(4.09-4.29) | 4.98(4.95-5.00) | 5.00(5.00-5.00) | 4.49(4.37-4.62) |
|  | 5 | 4.29(4.17-4.40) | 4.25(4.15-4.36) | 4.97(4.15-4.36) | 5.00(5.00-5.00) | 4.63(4.52-4.75) |
|  | Pooled | 4.27(4.22-4.32) | 4.23(4.18-4.27) | 5.00(5.00-5.00) | 4.97(4.95-4.99) | 4.58(4.53-4.64) |

# **Appendix E**

**Table E.6. The results of the ablation experiments on the Qwen72B model.**

|  | BERTScore | Embedding Avg Scores |
| --- | --- | --- |
| LLM | 43.66 (34.43-49.76) | 88.30 (78.66-90.70) |
| LLM+RAG | 43.84 (36.88-51.34) | 88.09 (79.03-90.19) |
| LLM+Search | 44.82 (36.91-50.81) | 87.11 (78.95-89.54) |
| LLM+RAG+Search | 44.88 (38.93-52.91) | 88.57 (84.21-91.30) |

# **Appendix F**

Here is a typical error example, which falls under the category of lifestyle-related issues.

Question: 慢性胃炎患者是否可能吃酸性水果，比如柑橘类？

Answer (7B-Agent): 慢性胃炎患者在饮食上应避免食用酸性水果，如柑橘类、葡萄、柚子、桔子、柿子、猕猴桃等。这些水果可能刺激胃酸分泌，加重胃部不适。建议慢性胃炎患者以清淡饮食为主，避免辛辣、生冷、刺激性食物，同时注意定时定量进食。在选择水果时，应优先考虑碱性水果，如柿子、柑橘类等，以帮助中和胃酸。对于急性胃炎患者，在出现腹痛、腹胀、反酸、烧心等症状时，应避免食用任何水果。在饮食调理的同时，还需注意避免腌制食物和油炸食品，以减轻胃部负担。

Answer (72B-Agent): 慢性胃炎患者在选择水果时应谨慎，尤其是对于酸性水果，如柑橘类。在病情稳定、胃部不适症状不明显时，可以适量食用，但应避免过量，以防刺激胃黏膜，加重炎症。建议选择成熟的、酸度较低的水果，并在餐后食用，以减少对胃部的直接刺激。如果患者有胃酸过多的情况，更应限制酸性水果的摄入，以免加重症状。在饮食调理上，应以清淡、易消化的食物为主，避免辛辣、油腻、生冷及过于酸甜的食物，以保护胃黏膜，促进胃部健康。

Standard answer: 酸性水果（如橙子、柠檬等）可能加重胃酸分泌，刺激胃壁，因此不建议慢性胃炎患者大量食用酸性水果，需要适量或谨慎食用。特别是在病情有明显症状时，应避免食用这类高酸性水果，以免刺激胃黏膜，加重胃部不适。慢性胃炎患者在饮食上应遵循“适量、温和”的原则，避免食用过于刺激的食物，包括高酸性的水果。

Expert opinions: The AI-generated response contains internal contradictions and misclassifies the acid–base properties of certain fruits. Persimmons must be consumed with numerous precautions even by healthy individuals; for patients with chronic gastritis they are particularly high-risk. Recommending that this population eat persimmons is therefore medically unsafe.
